# Design and Biological Evaluation of Delivery Systems Containing Bisphosphonates

**DOI:** 10.3390/pharmaceutics9010002

**Published:** 2016-12-26

**Authors:** Blessing Aderibigbe, Isiaka Aderibigbe, Patricia Popoola

**Affiliations:** 1 Department of Chemistry, University of Fort Hare, Alice Campus, Eastern Cape 5700, South Africa; 2 Department of Chemical and Metallurgical Engineering, Tshwane University of Technology, Pretoria 0001, South Africa; ia.aderibigbe@gmail.com (I.A.); popoolaapi@tut.ac.za (A.P.)

**Keywords:** bisphosphonates, drug delivery systems, biomaterials, nanocapsules

## Abstract

Bisphosphonates have found application in the treatment of reoccurrence of bone diseases, breast cancer, etc. They have also been found to exhibit antimicrobial, anticancer and antimalarial activities. However, they suffer from pharmacological deficiencies such as toxicity, poor bioavailability and low intestinal adsorption. These shortcomings have resulted in several researchers developing delivery systems that can enhance their overall therapeutic effectiveness. This review provides a detailed overview of the published studies on delivery systems designed for the delivery of bisphosphonates and the corresponding in vitro/in vivo results.

## 1. Introduction 

Osteoporosis, osteoarthritis and bone cancer are the most commonly diagnosed skeletal disorders [1], while osteoporosis has been identified as the major cause of bone fractures globally and the most frequently diagnosed among bone diseases. Overall, studies have shown that over 200 million people suffer from the disease [2,3]. Annually, osteoporosis is responsible for more than 8.9 million fracture cases, accounting for an osteoporotic fracture every 3 s [4]. It is a progressive disease that causes decrease in bone mineral density. Osteoporosis is commonly found in older postmenopausal women [5]. Overall, prevalence in women increases significantly with age, from 2% by age 50, and up to 25% at age 80 [6]. In general, estrogen is reduced by menopause, leading to increased bone resorption [7,8]. Thus, indicating that major additive factors of osteoporosis are menopause and advancement in age. In addition, other diseases such as spinal cord injuries and rheumatic arthritis, which affect the bone turnover, may result in osteoporosis [9]. It is significant to note that solid tumors such as lung, prostate and breast cancer, and multiple myeloma are also susceptible to develop bone diseases [10]. Osteoporotic bones are thinner, smaller and characterized by increased brittleness and high porosity [11]. 

In general, disruption in bone micro-architecture features leads to reduction in bone mineral density relative to normal healthy bone. Osteoporotic bone exhibits reduced collagen, and consequently results in loss of protein which leads to weakening of bone [12]. Thus, osteoporotic bone is very susceptible to fracture due to increased fragility [6,13]. Fractures have been located in the wrist, proximal humerus, vertebrae and the spine of osteoporotic patients [14,15,16]. In general, potential risks associated with osteoporosis can be minimized by engaging in regular exercise with healthy life style, and consumption of calcium and vitamin D enriched diets [17].

Clinically, bisphosphonates are the most widely administered therapeutic agents for treating conditions with increased bone resorption associated with osteoclastic activity, as well as osteoporosis, Paget and metastatic bone diseases [18,19]. Bisphosphonates disrupt bone resorption and lead to osteoclasts death, thereby reducing loss of bone mass [20]. The most commonly used bisphosphonates due to their effectiveness for bone metastasis therapeutic include: zoledronic acid, pamidronate, clodronate and ibandronate, etc. [21,22,23]. However, in spite of their pharmaceutical efficacy, they suffer from a few drawbacks which include: poor oral bioavailability (1%–3%) [24,25,26], side effects of acute gastro-intestinal conditions such as gastric ulceration, dysphagia and esophagitis [27,28]. In order to enhance the bioavailability of bisphosphonates, several efforts have been made, such as improvements in the design of drug delivery systems, use of absorption enhancers and structural modification of the drugs [29,30,31,32,33]. On the other hand, infected site targeting controlled release of bisphosphonates has the capacity to enhance effectiveness of drugs and minimize the associated side effects [34,35,36,37]. This ameliorates the inconvenience and the patient morbidity related with musculoskeletal disorders.

## 2. Classification of Bisphosphonates

**Figure 1:**
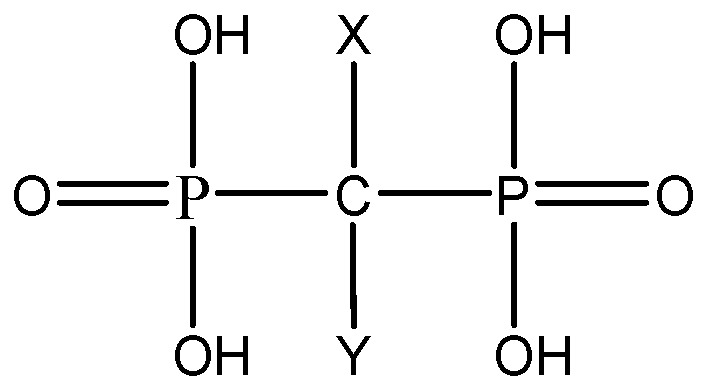
Schematic representation of bisphosphonates.

Bisphosphonates are classified as stable pyrophosphonate analogues [38]. They belong to a group of drugs distinguished by a P–C–P bond covalently attached two side chains, X and Y, the central oxygen atom being substituted by a carbon atom [39,40] (Figure 1). 

The presence of nitrogen on the Y chain results in nitrogen-containing bisphosphonates (N-BPs). On the other hand, when nitrogen is absent, the compound is referred to as non-nitrogen-containing bisphosphonates (non N-BPs) [41,42,43,44,45]. When the hydroxyl (OH) or amino (NH_2_) groups are present as substituents to X, they enhance the bone binding properties of bisphosphonates through tridentate binding to calcium, while the major function of Y is to influence bisphosphonates’ antiresorptive potency [46]. It is worthy to note that though the hydroxyl and phosphate groups are significantly responsible for the bisphosphonates’ attraction for the bone matrix, however, the final structural moiety (in the Y position) which bounds to the carbon at the center mainly determines the efficacy of bisphosphonates to inhibit bone resorption [47]. 

**Figure 2:**
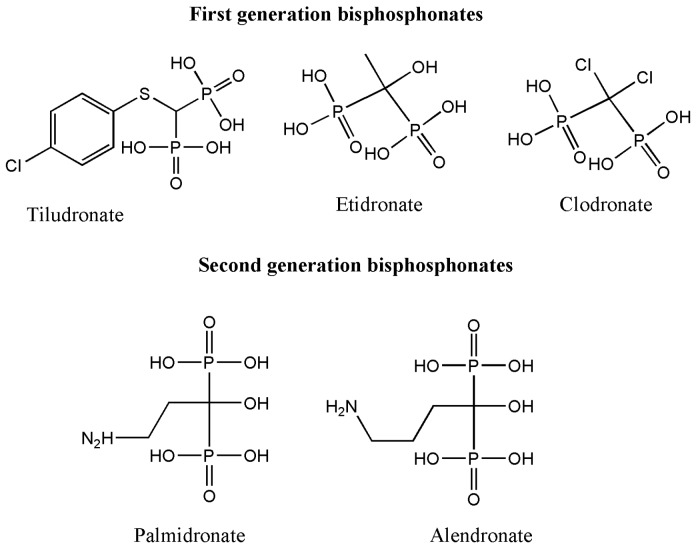

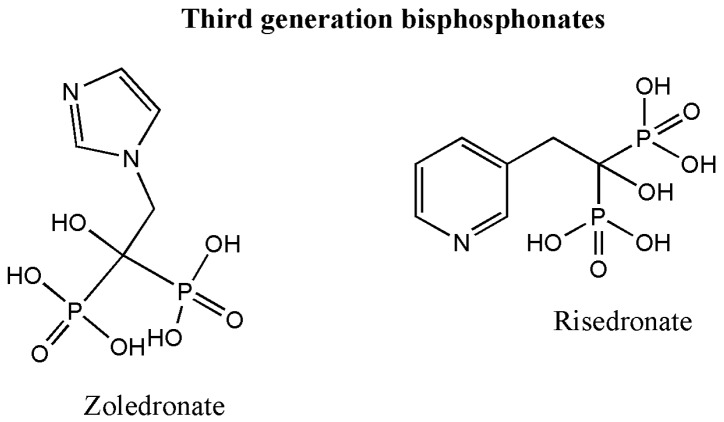
First, second and third generation bisphosphonate.

Bisphosphonates are also classified into three generations (Figure 2): a first generation consisting of clodronate, tiludronate and etidronate, a second generation consisting of pamidronate plus alendronate, while the third generation consists of zoledronic acid plus risedronate [47]. The efficacy of bisphosphonates arguably has been improved between 10 and up to 10,000 times compared to bisphosphonates which do not contain nitrogen, i.e., etidronate [48,49].

## 3. Mechanism of Action of Bisphosphonate

The mechanism of action of bisphosphonates has been described as complex and involving several factors [50]. The effective mechanism is traceable to their identical structures to that of pyrophosphate [51]. In spite of the complexity of the mechanism of action of bisphosphonates, the binding attraction (R1 side-chain) as well as the antiresorptive potency (R2 side-chain) are both controlled by the side chains. The drugs have an exceptional attraction for the bone hydroxyapatite (HAP), resulting in bisphosphonates being deposited in close proximity to osteoclasts in newly formed bones [50]. During resorption phase, osteoclast microenvironment (endosomes) is vastly acidic, which may promote bisphosphonates release from the bone surface, leading to an increased bisphosphonate local concentration on the bone mineral surface [52], especially at the osteoclast activity site, by rapid clearance from the systemic circulation [53]. Various types of bisphosphonates have unique antiresorptive potency and binding affinities. Their efficacy increases in the following order: clodronate < etidronate < risedronate < ibandronate < alendronate < zoledronate [50]. The activity of mononucleated osteoclasts, the parent cells of osteoclasts, is affected directly once bisphosphosnates enter into the bone [50], resulting in the disruption of osteoclast intervention in the bone resorption and a rise in osteoclast apoptosis, which finally leads to bone deposition by osteoblasts. The resultant effect is the reduction in bone resorption and turnover [50].

It is worthy to note that each bisphosphonate has a unique mechanism of action [54]. The metabolic by-products of non-nitrogen containing bisphosphonates are toxic and lead to cell death (cellular apoptosis). On the other hand, nitrogen containing-bisphosphonates bind and inhibit the enzyme farnesyldiphosphate synthase, thus resulting in reduced cell functions and cell death [55,56,57]. Non-nitrogen-containing bisphosphonates are prescribed less often to patients because they exhibit more detrimental effects than the nitrogen-containing type [58].

## 4. Application of Bisphosphonates

Bisphosphonates effectively inhibit bone resorption by osteoclasts, it has also been suggested that they may initiate the rapid multiplication of osteoblast bone-building cells [59,60,61,62]. As a result, the drugs have found a wide range of medical applications which include their uses in the treatment of Paget’s disease, osteoporosis prevention and treatment, hypercalcaemia or non-hypercalcemia related bone metastasis, primary hyperparathyroidism, multiple myeloma, osteogenesis imperfecta, fibrous dysplasia and other bone conditions characterized by bone frailty [63,64,65].

### 4.1. Bisphosphonates in Osteoporosis

Osteoporosis is a medical condition whereby a reduction in bone mass leads to bone fragility which may increase fracture tendencies. The processes of bone formation and resorption are closely linked under normal bone development. On the other hand, in osteoporosis, the net rate of bone formation is lower than that of bone resorption, a condition which leads to bone mass reduction without a defect in bone mineralization [66]. It is a common practice to treat osteoporosis with bisphosphonate, particularly post-menopausal osteoporosis and idiopathic osteoporosis commonly found in men, secondary osteoporosis associated with different diseases and glucocorticoid-induced osteoporosis [67,68,69].

### 4.2. Bisphosphonates in Orthopedic Medicine

Orthopedic surgery is the branch of medicine concerned with conditions involving the musculoskeletal system. The surgeon employs both non-surgical and surgical methods to treat musculoskeletal trauma, tumors, spine diseases, injuries, degenerative diseases, infections, etc. [70]. The number of skeletal disorders affecting bone mineral density is very significant; this has been attributed to the increased osteoclastic activity. Some diseases which include skeletal metastatic and osteoporosis are prevalent in the general population while other diseases such as Paget’s disease and osteogenesis imperfecta are less frequent [71]. The primary reason bisphosphonate is used in orthopedics is to improve the bone mass in addition to preventing the possibility of fracture especially in osteoporosis patients [72], and to prevent bone collapse in the event of worst case scenario of osteonecrosis [50].

### 4.3. Bisphosphonates in Paget’s Disease

Paget’s disease affects bone development and how it renews itself, resulting in the affected bone becoming unusually weak. The common complaint among patients is pain in the affected bone area and sometimes elsewhere, while other patients have reported no symptoms. The commonly affected bones are spine, thigh, shin and pelvis. In time, a number of complications could develop, such as bending, nerve compression, osteoarthritis, deafness, bone enlargement, fracture while tumors have been observed in rare cases. A common therapy for the condition is through prescription of painkillers in combination with bisphosphonates, which may be administered as tablets or infused directly into the blood stream [73]. 

### 4.4. Bisphosphonates in Oncology

Oncology is a branch of medicine that specializes in the prevention, diagnosis and therapy of cancer. In cancer patients, especially those diagnosed with prostate, lung or breast cancer, the development of bone metastasis is very common [74,75]. Various skeletal disorders have been observed to be prevalent in cancer patients with bone metastasis, which include serious hypercalcaemia or severe bone pain and unexpected pathological fractures [74]. Presently, the best known first-line treatment for bone metastasis are bisphosphonate drugs [74,76,77] due to their efficacy in reducing pain and fracture tendencies [78], in breast cancer patients [79], lung cancer [80], and in multiple myeloma as well as other cancers patients [81]. 

### 4.5. Administration Routes of Bisphosphonates

Bisphosphonates are administered in the therapy of metastatic bone diseases via oral or intravenous routes [82]. Apart from intravenous and oral routes for the administration of bisphosphonates, a nasal route has also been reported. The nose offers access to mucosal surfaces suitable for the delivery of vaccines and bioactive agents. This route offers several advantages: it provides a direct route for drugs into the blood stream; it protects the drugs from enzymatic attack that is common with oral administration of drugs resulting in enhanced bioavailability; the rate of absorption and plasma concentration is better than the subcutaneous routes; it is convenience, easy and painless [83]. Cruz et al., prepared spray-dried powders for delivery of sodium alendronate to the lungs. The formulation particle size was below 12 μm. The administration of sodium alendronate dry powder did not induce significant lung toxicity [84]. Sutton et al. reported the nasal absorption of alendronate in dogs and rats which was higher than the peroral route [85]. 

Bisphosphonates can also be delivered through the pulmonary route. In pulmonary delivery of drugs, it can be administered by two ways: by intranasal and by oral inhalative administration [86]. Oral inhalative administration can also be classified as intratracheal instillation and intratracheal inhalation [86]. Ueno et al. reported that inhalation of alendronate induced apoptosis in alveolar macrophages, a pathogenesis of emphysema [87]. Katsumi et al., examined the absorption of alendronate via intrapulmonary administration in rats. The bioavailability of alendronate after intrapulmonary administration was more enhanced than oral administration. Intrapulmonary administration in rats with 1α-hydroxyvitamin-D_3_-induced hypercalcemia suppressed significantly the decrease in bone mass in a rat model of osteoporosis [88]. Katsumi et al., developed polyethylene glycol-conjugated alendronate, a novel and evaluated the absorption of the formulation in rats via intrapulmonary administration. The bioavailability of the formulation was similar to the oral administration of alendronate. There was evidence of damage of the pulmonary epithelium by the intrapulmonary administration of alendronate. In an osteoporosis model in rats, intrapulmonary administration of PEG–alendronate inhibited a decrease in the width of the growth plate similar to oral administration [89]. In an invention by Bhatnager et al., nanosize bisphosphonate particles in form of dry powder inhaler or through nebulizer for intrapulmonary administration. The formulation exhibited a sustained delivery mechanism of the drug to the lungs over a period of 24 h [90].

The vagina is a route for administration of drug systems such as contraceptives, anti-fungals and antimicrobials. This route offers several advantages such as prolonged release, increased drug bioavailability, ease of use, reduction of drug degradation by enzymes, etc., quick onset of drug action and reduced side effects [91]. In an invention formulations for vaginal targeted delivery of bisphosphonate drugs were prepared. The formulations delivered the bisphosphonates 10 to 30 times better, compared to oral administration and was able to overcome problems associated with oral administration such as gastric and esophageal reflux and ulceration [92]. The formulations were potential drugs for the treatment of osteoporosis, Paget’s disease, metastatic cancer of bone, and other related diseases of bone [93,94,95]. Ozdemir et al., compared the bone uptake of alendronate sodium from vaginal suppositories prepared with massa estarinum and polyethylene glycol 1500 (PEG) bases. The suppository base was useful in drug release [96].

## 5. Types of Systems Used for Delivery of Bisphosphonates

In general, various drug delivery systems can be classified into two major groups: biodegradable and non-biodegradable. Biodegradable drug delivery systems are specific delivery systems which break down within the body after natural biological processes. On the other hand, non-biodegradable drug delivery systems are those delivery systems which do not break down after the active agents have been released, thus necessitating the removal of such delivery systems from the body. It is worthy of mention that all the biomaterials reported by the researchers for the delivery of bisphosphonates were claimed to be biodegradable after modifications.

However, systemic administration of the drug can produce some unpleasant side effects such as ocular inflammation, electrolyte imbalance and acute systemic inflammatory reactions, etc. [82]. Thus, it is pertinent to devise therapeutic methods of achieving less toxicity, improved efficiency and better drug delivery. Targeted delivery systems are emerging and promising technologies for delivering drugs to the required skeletal pathological sites which exhibit improved potentials in reducing toxicity to the targeted sites. The targeted delivery systems improve the solubility of the drugs and also ensure that the drugs are not degraded or eliminated in the blood circulatory system [97,98]. Ideal properties of a drug delivery system should include timely release and good control rate of drugs at the required sites through the physical and chemical characteristics of the carriers. Numerous delivery systems for bisphosphonates have been explored [99,100,101,102,103,104,105,106]. Due to the poor oral bioavailability of bisphosphonates, various strategies as well as delivery techniques have been attempted, such as polymers conjugates, hydrogels, liposomes, bioceramics, nanocapsules, nanospheres, etc. [99,107,108,109,110,111,112,113,114].

### 5.1. Polymer Drug Conjugates (Copolymers)

**Figure 3:**
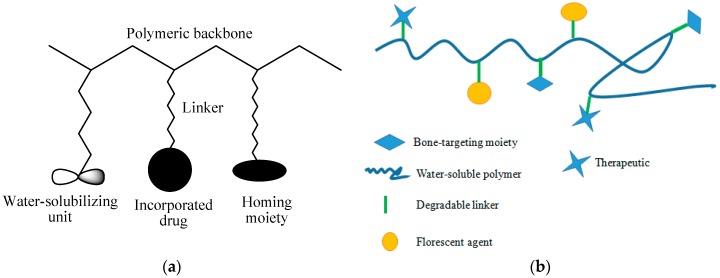
(**a**) Schematic representation of polymer-drug conjugate; (**b**) a schematic diagram of polymer-drug conjugates containing bioactive agents.

Polymers refer to materials which exhibit high molecular weight and consist of long chain-like molecules with repeating units (monomers) of identical structure. They can either be naturally occurring e.g., gelatin, albumin starch fibrinogen, dextrose or synthetic i.e., poly(dl-lactic acid) poly(glycolic acid) and poly(l-lactic acid), etc. [115]. Although natural polymers can be used as drug conjugates, however, there are some drawbacks to their usage. Some natural polymers, particularly gelatin and starch, exhibit many biodegradable bonds, which are easily degraded in a biological environment. Thus, modification of their structures is necessary to reduce the rate of degradation. In addition, they also suffer from a limited number of functional groups suitable for drug binding [116]. Polymer drug conjugates consist of polymer backbone, linker, solubilizing unit, homing moiety, and incorporated drug (Figure 3a).

Polymeric therapeutics or drug delivery systems are nano-sized water soluble polymers to which drugs are covalently bound. They are uniquely characterized by effective pharmacological and pharmacokinetic properties [117]. They include dendrimers, liposomes, nanospheres, nanocapsules and polymer-drug conjugates [117]. Drugs are loaded onto the polymers using known techniques which have been approved for therapeutic applications. Their benefits include: enhanced drug bioavailability, enhanced water solubility, reduced drug resistance, non-antigenic, reduced drug toxicity, prolonged plasma half-life which results in reduced kidney clearance, non-immunogenicity, protection of drugs from degradation by inhibiting enzymes, versatile applications in multiple-drug delivery, ability to accumulate in specific organs, tissues or cells by improving the effect of retention and permeability [116]. On the other hand, biodegradable synthetic polymers have become more attractive alternatives due to [118]: (i) variety in material properties and if properly designed; there is possibility of further modifications without altering material bulk properties (ii) the synthetic polymer structure is easily controlled; and (iii) biomimetic synthetic polymers usually exhibit drug delivery efficacy and biocompatibility. Polymers possess high capacity to absorb water to various levels, sometimes exceeding 90% weight subject to the polar functional group type present in the network structure. However, it should be noted that the rate at which polymers absorb water is influenced by their compositions and the aqueous environment [119].

Polymer-drug conjugates have been employed for incorporation of bisphosphonates (Figure 3b) (Table 1). Aderibigbe et al. reported polyamidoamine conjugates of neridronic acid that were found to be potential prodrugs [120]. Polyhydroxyaspartamide-based conjugates containing bisphosphonate derivatives was prepared by Paolino et al., [121]. The results of the in vivo tests performed on rats indicated that the conjugates demonstrated selective behaviour towards bone tissues, indicating that they are potential drug delivery systems for bone tissues therapeutics. In another study, drug release studies and characterization of polyamidoamine conjugates containing curcumin and bisphosphonate were conducted by Aderibigbe et al., who used a one-pot aqueous phase Michael addition reaction resulting in the bisphosphonate forming an integral part of the polymer carrier backbone [120]. A polymer-drug conjugate of alendronate from poly(lactide-*co*-glycolide) based delivery system was prepared by Pignatello et al. The carriers were observed to be suitable as drug delivery systems to bone tissues [122].

In a research report by Pan et al., Hydroxypropyl methacrylamide (HPMA)-alendronate conjugates were prepared and the results of in vivo tests performed by administration of radio-iodinated conjugates to young healthy BALB/c mice (albino, laboratory-bred strain of the house mouse) through an intravenous route suggested that the biodistribution of the conjugates in mice was as a result of the strong binding capacity of the conjugates to the bone. It is significant to note that alendronate content in the conjugates exhibited no effect on bone deposition capacity. On the other hand, the bio-distribution of the conjugates was influenced by the molecular weight [123]. Miller et al., utilized a bone-targeting moiety in old, ovariectomized rats. They reported HPMA copolymers absorption as well as localization in rats by attaching protogladin E_1_ to HPMA copolymer-octapeptide conjugate through a cathepsin K-sensitive linkage. They administered a dose of the conjugate by injecting the rats, which resulted in improved bone formation [124]. In another report by Hruby et al., it was claimed that the drug release of in vitro studies of biocompatible poly[*N*-(2-hydroxypropyl)methacrylamide] carrier which contains hydroxybisphosphonate targeting moieties plus the model radio therapeutics was dependent on enzymatic stimulus and pH [125]. The pharmacokinetics and bio-distribution of HMPA copolymers in young, healthy BALB/c mice was investigated by Wang et al. It was found that the HPMA copolymers were deposited on the entire skeleton, which was facilitated by the conjugate higher molecular weight which was attributed to the extended half-life during circulation. However, they reported that this resulted in poor bone selectivity [126]. Earlier, Wang et al., had reported the development of cathepsin K inhibitor-polymer conjugates using HPMA carriers. It was suggested that the polymeric inhibitors accumulated around the active bone resorption site, where it was absorbed by osteoclasts followed by antiresorptive activity [127].

### 5.2. Hydrogels

**Figure 4:**
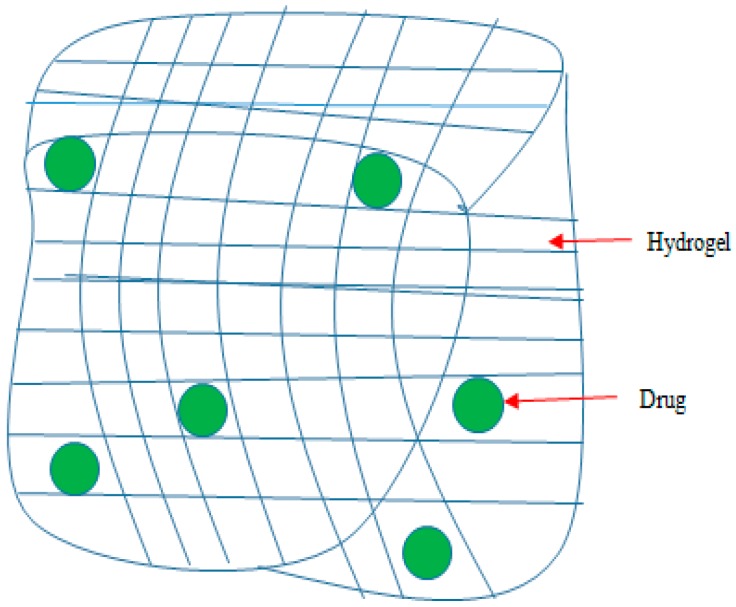
A schematic diagram of hydrogel loaded with bioactive agent.

Hydrogels belong to a group of polymeric materials with high capability to absorb fluids, e.g., water or biological fluids, in large amounts (Table 2). Their polymeric structures contain: –CONH–, –SO_3_H, –CONH_2_– and –OH hydrophilic functional groups, which react to form hydrogels [119]. They consist of porous structures that facilitate the drug uptake and release. The porosity of the hydrogel can be adjusted either by decreasing or increasing the degree of the gel matrix cross linking, which controls the level of swelling in aqueous medium [120]. Polymeric materials (i.e., hydrogels) which include poly(lactide-*co*-glycolide) or poly lactic acid (PLA) with hydrophobic characteristics exhibit low water-absorption capacity behavior (5% ≤ *x* ≤ 10%). This is attributed to their inherent high water content which influences their physico-chemical characteristic. The physico-chemical properties of hydrogels have been described as being identical to those of human tissue [119]. They exhibit two types of crosslinking: (i) physical crosslinking, involving entanglement of crystallites; and (ii) chemical crosslinking involving tie points and junctions. Factors responsible for the crosslinking of structures in hydrogels include: hydrogen binding, van der Waals interactions, covalent bonds, or physical entanglements [128,129]. Hydrogels are insoluble in water due to the presence of chemical crosslinks (Figure 4) [130]. Recently, there has been increasing special interest towards natural polymers containing hydrogels due to their biodegradability, hydrophilic, non-toxic and biocompatibility characteristics, leading to their significant applications in the biomedical field [131]. Gum acacia is a good example of a natural occurring polysaccharide. It is biodegradable, soluble in water, non-toxic, pH stable, readily available and environmentally friendly [120]. It is derived from (1→3) and (1→6)-linked β-d-galactopyranosyl units in conjunction with (1→6)-linked β-d-gluco-pyranosyluronic acid. The side branches consist of α-l-rhamnopyranose, β-d-galactopyranose, α-l-arabinofuranosyl units, and β-d-glucuronic acid with (1→3), (1→4), and (1→6) glycosidic linkages [132]. Aderibigbe prepared gum acacia-based hydrogels and incorporated bisphosphonate. The hydrogels were found to be promising systems for the delivery of bisphosphonates to the gastrointestinal region [133].

Hulsart-Billström et al. developed a functionalized hyaluronan-based hydrogel with covalently linked bisphosphonate ligands incorporated with bone morphogenetic protein-2. The hydrogel released less than 10% of the bone morphogenetic protein-2 over a period of two weeks. The successful entrapment of bone morphogenetic protein-2 in the hydrogel preserved the growth factor bioactivity, confirming the induction of osteogenic differentiation of mesenchymal stem cells subsequent to the incubation of cells with the hydrogel enzymatic digest. Osteoblasts were not affected by the products’ degradation [134]. Khajuria et al. developed pH-sensitive photonic composite hydrogel beads comprising risedronate sodium and sodium alginate [135]. The hydrogel beads’ progressive release ratios of risedronate through the composite were 2.47% in pH 2.1 solution and 83% in pH 6.8 solution over a period of 24 h. The system exhibited a potential to increase the intestinal absorption of risedronate [135].

Kettenberger et al. combined zoledronate and nanoparticles of hydroxyapatite in order to peri-implant bone reinforcements. Hyaluronic-based hydrogels loaded with zoledronate or hydroxyapatite nanopartlcles were introduced by injection into rat femoral condyles via predrilled screw holes. The hydrogels exhibited rapid mineralization with the formation of granules serving as new bone formation scaffolds, suggesting they are effective bone repair materials [136]. Pasadowska et al. loaded alendronate onto gellan gum based hydrogels which released the drug over a period of 25 days. The loaded hydrogel was cytocompatible with MG-63 osteoblast-like cells and it inhibited RAW264.7 cells’ osteoclastic differentiation mediated by RANKL [137]. Kootala et al. developed a hyaluronan-based hydrogel functionalized with bisphosphonate groups. The loaded hydrogels allowed enhanced systematic release of bone morphogenetic protein-2, which was dependent on the number of bisphosphonate groups introduced to the hydrogel [138].

### 5.3. Bioceramics

Despite the popularity and wide use of hydrogels and biodegradable polymers for biomedical applications and the engineering of cartilage and bone [139], their applications are limited because they are not suitable for use at load-bearing sites of the body [140]. Tolerable alternative materials for therapeutics are natural and synthetic ceramic materials [141,142]. Ceramics are non-metallic inorganic materials, they are hard and brittle and form a group that include crystalline, amorphous and glass ceramics. Examples of bioceramics include calcium phosphates, bioactive glasses and alumina [143]. In orthopaedic surgery, ceramics can be grouped into various sub-divisions according to their bioactivity (in vivo reactivity), load bearing capacities and surface chemistry properties i.e., whether they are biodegradable or bioinert (Table 3) [144]. In orthopaedics, bioceramics can be further divided into two large sub-groups, which include: (i) calcium phosphate-based ceramics (i.e., hydroxyapatite) used for bone regeneration and (ii) high strength ceramics which are applied in load bearing sites, e.g., zirconia—an important ceramic used in hip prostheses and ball heads in dental caps [144].

Hydroxyapatite (HA) is bioactive ceramic; it is a suitable bone replacement material as it is regarded as a mineral component of the bone [145,146,147] and provides sufficient mechanical properties. Hydroxyapatite (HA) and other bioactive ceramics, i.e., tricalcium phosphate (TCP), silicate and phosphate glasses (bioactive glasses) consisting of certain chemical compositions form tight bonds with hard tissue through cellular activity and by reacting with physiological fluids [143]. When metal implants are coated with hydroxyapatite (HA), it results in enhanced in vivo bone integration [148,149,150,151]. In vitro comparison between hydroxyapatite culture surfaces to titanium and glass-ceramic indicated that culture surfaces of hydroxyapatite exhibited enhanced rat marrow stromal cells’ differentiation compared to osteoblasts [152]. In general, a promising method to enhance biomaterial osseointegration is by using surface modifications like adhesive peptides in order to control the interactions at the interface of bone-implant in order to facilitate osteoprogenitor cells adhesion, or the local delivery of growth factors, which in turn will lead to stimulation of cell differentiation and promote healing and fixation [140].

In view of the disease transfer risks and immunological concerns from allogeneic bone, extensive research work has been devoted to the development of ceramic-based alloplastic bone substitutes that are predominantly based on ceramic materials which include calcium phosphates (CaP), calcium sulfates, and bioactive glasses [141]. These ceramic materials are generally popular due to their bioactive and osteoconductive properties [142]. CaP-based ceramics hydroxyapatite (HA) and beta tricalcium phosphate are the most commonly used ceramic materials [144].

In a study by Balas et al. MCM-41 and SBA-15 mesoporous matrices were loaded with alendronate [153,154], under similar conditions, with a maximum drug loading of 14% for MCM-41 and 8% for SBA-15. Drug loading was increased to 37% and 22%, respectively for functionalised MCM-41 and SBA-15. Drug release after 24 h showed that there was an increase in the total drug delivered from functionalized materials when compared to the unmodified matrices [153,154]. In a study by Denissen et al. ceramic hydroxyapatite implants were prepared for the delivery of bisphosphonates in order to maintain bone mass after extraction of teeth [154,155]. Four different types of ceramic hydroxyapatite implants were designed and assayed in saline at an ambient temperature of 37 °C over a 3-month period. The release of bisphosphonates from the ceramic hydroxyapatite was steady and controlled, suggesting that they were potential release systems for bisphosphonates [143]. Sorrensen et al. developed calcium phosphate-like bone substitute materials loaded with zoledronic acid by a dipping technique and the release of zoledronic was controlled [156]. 

### 5.4. Hybrid Compounds 

Hybrid compounds are produced by combining two dissimilar and independently functioning compounds at the molecular or nanometer level to form a covalently-linked hybrid compound which can produce interactionw from the individual effect of the two independently acting moieties to the newly formed composite compound, resulting in a higher pharmacological efficacy compared to the sum of each moiety’s efficacy [157]. There are few reports on hybrid compounds containing bisphosphonates (Table 4).

Yao and Lane, developed a hybrid compound, LLP2A-Ale in which the constituent LLP2A has high affinity for the α4β1 integrin on mesenchymal stem cells (MSCs) and alendronate—a bisphosphonate—has high affinity for bone. They injected LLP2A-Ale into mice and found that the compound directed MSCs to both cortical and trabecular bone surfaces with improved bone strength and bone mass [158]. Bekker et al. prepared hybrid compounds of bisphosphonate containing folic acid and bisphosphonate [159]. Yang et al. prepared and investigated the effect of the conjugate on growth inhibition and apoptosis in human osteosarcoma MG-63 cells [160]. El-Mabhouh et al. developed a hybrid compound containing gemcitabine, an anticancer drug and a bisphosphonate molecule [161]. Nakatake et al. prepared platinum complexes containing bisphosphonate. The complexes were evaluated as metastatic bone tumor and the complexes exhibited stronger tumor growth inhibitory effects than cisplatin [162]. 

## 6. Carbon-Based Materials

### 6.1. Carbon Nanotubes 

Carbon nanotubes are carbon nanomaterials with low-dimensional sp^2^ bonding and distinguished by some unique chemical and physical characteristics with various potential uses in many areas, including nanomedicine [163]. They are carbon allotropes consisting of graphite sheets rolled up into cylindrical tubes. Two types of carbon nanotubes are available: (i) single-walled nanotubes, which are characterized by a single graphene sheet with varying geometry of 20–1000 nm in length and 0.5–3 nm in diameter; and (ii) multi-walled nanotubes produced from several concentric graphene sheets, also exhibiting varying geometries of 1–50 micron in length and 1.5–100 nm in diameter [164]. They are reputed as excellent drug-delivery carriers by directly entering into the cells and sustaining drug efficacy without metabolism during transport in the body (Table 5) [165,166,167]. The mechanism of carbon nanotube drug delivery consists of attaching a drug inside functionalized carbon nanotubes or on the surface. The conjugate is then conventionally administered to the animal using either an oral route or injection. It can also be directly targeted at the required organ site using magnetic conjugates. The carbon nanotube drug conjugate is then engulfed by the cell after which the nanotubes deliver the drug at the target organ [166,167,168,169,170]. Despite all the advantages of carbon nanotubes, there are drawbacks to their potential clinical implementations due to their intrinsic toxicity and poor bioavailability, which pose as challenges in clinical therapeutics. 

Mbianda et al. reported the conjugation of double wall carbon nanotubes with bisphosphonates for targeted passive accumulation via enhanced permeability retention (EPR) effect with reduced toxicity [171].

### 6.2. Fullerenes

**Figure 5:**
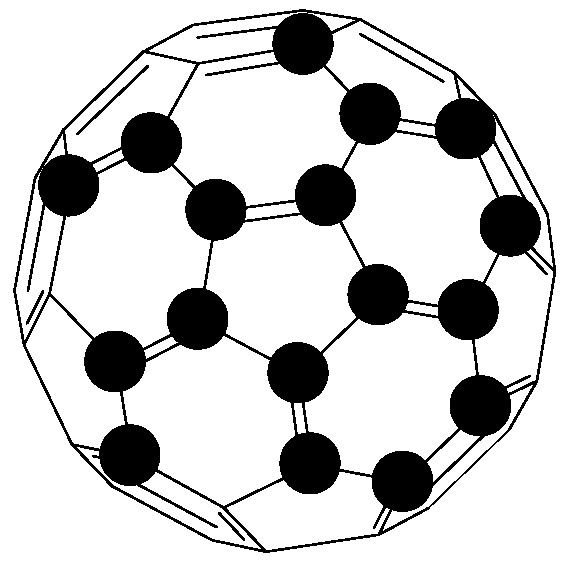
Fullerene structure.

Fullerenes are a group of allotropes of carbon with cage-like fused-ring structures which resemble a football or tubes (Figure 5). The behaviour of fullerenes is governed by both the core properties and the core chemical modification [173]. The functional group attached to the core is responsible for the complex behaviour of fullerene while the core is hydrophobic [173]. However, attachment of hydrophilic moieties to fullerenes enables them to be water-soluble with the capability of carrying genes and drugs for cellular delivery [173]. 

Foley et al., demonstrated that functionalized fullerenes are capable of crossing the cell membrane to bind with the mitochondria [174]. A bone tissue targeted bisphosphonate fullerenes C_60_(OH)_16_AMBP was prepared by Gonzalez et al. It was reported that through combination reactions of various hydroxyl group and an amide bisphosphonate addend resulted in achieving a strong attraction for calcium phosphate mineral HAP Ca_10_(PO_4_)(OH)_2_ in the bone [172].

## 7. Liposomes

**Figure 6:**
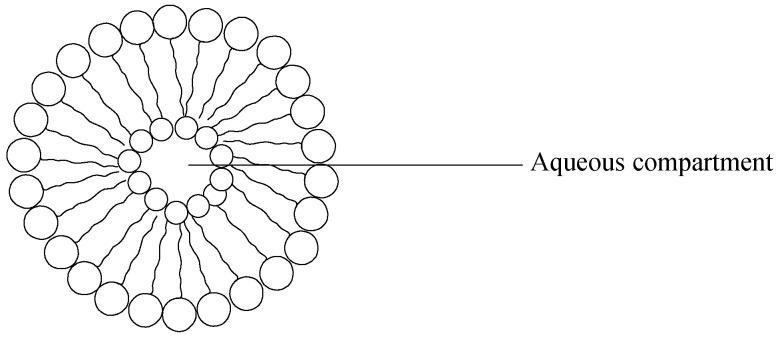
Structure of liposomes.

Liposomes are artificially prepared vesicles composed of natural phospholipids [115], with sizes varying between 20 nm and up to several microns [175]. The liposomes’ colloidal vesicles are self-assembled and made up of either single or multiple concentric lipid bilayers which consist of cholesterol and amphiphilic phospholipids, enclosing an aqueous compartment (Figure 6). Liposomes are versatile carriers as they can carry hydrophobic components in their lipidic bilayered membrane, and their internal aqueous core can also incorporate hydrophilic drugs, thereby preventing degradation of their payloads in the systemic circulation [176].

Liposomes exhibit an extremely flexible scaffold and their structural complexity affords a range of polar, non-polar and amphipathic drugs to be encapsulated (Table 6) [116]. A key factor in assessing liposomes’ circulatory half-lives is the vesicle size. The size and number of bilayers have also been found to affect the level of encapsulation of the drug within the liposomes [177]. Liposomes can further be divided into two groups: (i) unilamellar vesicles and (ii) multilamellar vesicles (MLV). Unilamellar vesicles can be subdivided further into: (a) small unilamellar vesicles (SUV); and (b) large unilamellar vesicles (LUV) [178]. Additional benefits of liposomal carriers besides their drug-loading capabilities include non-immunogenicity and good biocompatibility. Furthermore, the surface can be modified with polyethylene glycol (PEG) to improve the blood circulation time, and a fixed amount of drug delivery can be attained when functionalized with certain moieties [179,180].

Liposomes are promptly absorbed by phagocytic cells such as the spleen, liver and the avid reticulo-endothelial system, which is due to their lipid bilayers’ interaction tendency with cellular surfaces [181]. The benefits and drawbacks of liposome drug carriers are influenced by the physicochemical and colloidal characteristics such as the makeup, size, their natural signaling through the cell casings, stability and incorporation efficiency [182]. It is significant to note that coated liposome surfaces consisting a steric stabilization layer of a hydrophilic polymer such as polyethylene glycol may reduce cell uptake of the reticulo-endothelial system which consequently leads to extended circulation times compared to uncoated liposomes [116]. There are two drawbacks identified with the coated liposomes: it has been suggested that there is a high risk of accumulation of the polymer, which may cause impairment of cell on a long term basis because coated liposomes are not degradable by enzymes in mammals after cellular uptake [183]. Secondly, the polymer coatings in the target site result in the hindering of drug release and promote interactions of the target cell following the localization of liposome in the target region, thus reducing the drug efficacy. Another challenge that affects both coated and uncoated liposomes as reported by Romberg is that liposomes are generally observed to be responsible for the complement system activation in preclinical investigations, thus causing hemodynamic, adverse respiratory and hematological changes in the activating complement system which may result in hypersensitivity reactions in a clinical setting [184]. 

An amphipathic molecule containing a BP head group, 4-*N*-(3,5-ditetradecyloxybenzoyl)-aminobutane-1-hydroxy-1,1-bisphosphonic acid disodium salt, was prepared by Anada et al., and formulated into liposomes with cholesterol (CH) and distearoylphosphotidylcholine (DSPC). In vitro test results showed that the liposomes decorated with bisphosphonate moieties were observed to exhibit increased attraction for pure HA particles [185]. Hengst et al. incorporated liposomes with cholesteyl-trisoxyethylenebisphosphonic acid (CH-TOE-BP) modeled for mineral affinity. In vivo tests of the HA affinity potentials of the liposomes have shown promising results with HA particles [186]. However, their potency for mineral-binding in in vivo studies was not reported. A study conducted by Golomb involved the formulation of negatively charged liposomes after phagocytosis by macrophages/monocytes, the liposome lipid bilayers were disturbed by the lysosomal phospholipases present in the macrophage [187]. The dissolved bisphosphonate drug in the aqueous compartment is then released into the cell. However, the amount of released free bisphosphonate from dead macrophages or through leakage from liposomes was negligible and did not significantly affect cells’ metabolism. Golomb [187], suggested that highly endocytotic cells, such as human monocytes and RAW264 macrophages, and has shown that bisphosphonate encapsulation in liposomes improves their inhibitory behavior between 20 and up to 1000-fold in contrast with the free drug. Earlier, in another study conducted by Van Rooijen et al. [188], it was observed that depletion of macrophage and monocyte of the bone marrow, spleen and liver can be achieved by liposomal bisphosphonate (clodronate). Gabizon et al. invented liposomes comprising a membrane and an intraliposomal aqueous water phase composed of bisphosphonate together with an amphipathic weak base agent. The formulation was effective against tumor cell lines [189].

## 8. Micelles

**Figure 7:**
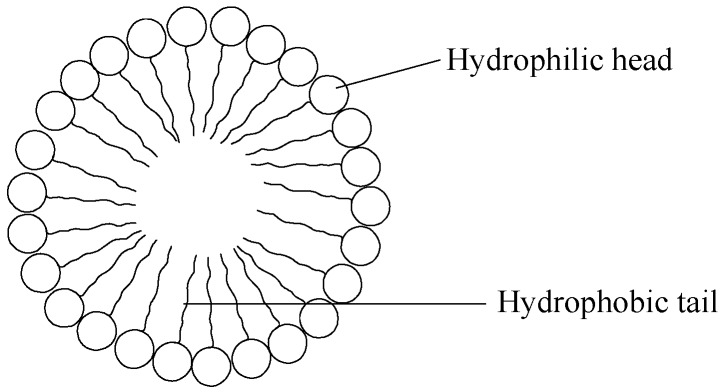
Structure of a micelle.

Micelles are nano-sized, supramolecular colloidal particles. They exhibit a hydrophobic core plus a hydrophilic shell produced by the self-assembling amphiphilic molecules aggregation, or surfactants in solutions (Figure 7) [190]. The intermolecular forces causing the segregation of the core segment from the aqueous environment can be used to classify block copolymer micelles. Three main groups of block copolymer micelles have been identified, which include: (i) amphiphilic micelles; (ii) polyion complex micelles, which were produced by hydrophobic and electrostatic interactions, respectively; and (iii) micelles produced from complexation of metals [191,192]. In general, spherical shaped micelles are formed when the core block is shorter than the hydrophilic segment. However, different non-spherical shapes, including lamellae and rods, may be produced when the core segment length is longer compared to the corona-forming chains [193]. The amphiphilic block copolymers’ self-assembly properties in water are attributed to non-polar and hydrophobic interactions between the lipophilic core-forming polymer chains. The self-assembly of amphiphilic block copolymers in water is attributed to the hydrophobic and non-polar interactions between the chains of the lipophilic core-forming polymer. The process is generally controlled by gain in entropy of the solvent molecules upon hydrophobic withdrawal from the aqueous media [194]. In drug delivery, most amphiphilic copolymers employed contain either a derivative of poly(amino acid) or a polyester as the hydrophobic segment [195]. Due to its excellent biocompatibility, poly(ethylene glycol) (PEG) is always used as the starting material to prepare hydrophilic block. However, hydrophobic block composition is designed to encapsulate drug molecules with a wide variety of charges, structures and lipophilicity, thereby enhancing the versatility of polymeric micelles as drug delivery systems. For polymeric micelles to be qualified for clinical applications, they must be water-soluble, biodegradable and biocompatible [190]. The block polymers which are frequently studied include: PEG-poly(ε-caprolactone) (PEG-PCL), PEG-poly(amino acids), PEG-poly(propyl oxide)-PEG (PEG-PPO-PEG, Pluronics), PEG-poly(d,l-lactide) (PEG-PLA) and PEG-distearoylphosphatidyl- ethanolamine (PEG-DSPE) [196]. Examples of micelle delivery systems are shown in Table 7.

Wang et al. prepared distearoylphosphoethanolamine-polyethylene glycol conjugate with 2-(3-mercaptopropylsulfanyl)-ethyl-1,1-bisphosphonic acid (thiolBP) which was incorporated into micelles and liposomes. It was prepared by the application of reverse-phase evaporation vesicle techniques and lipid film hydration methods. The designed liposomes were able to entrap the bone morphogenetic protein-2 in a bioactive form, suggesting their ability to deliver bioactive factors in mineralized scaffolds for bone tissue engineering [197].

Ye et al. prepared multifunctional micelles using doxorubicin-poly(ethylene glycol)-alendronate as amphiphilic material. They reported that doxorubicin-loaded micelle retarded tumor growth, reduced bone loss and decreased cardiac toxicity in tumor-bearing mice, suggesting their potential uses for treating bone metastatic tumor [198].

Miller et al conjugated alendronate and paclitaxel with poly(ethylene glycol) forming self-assembled micelles with paclitaxel molecules at the inner core and the alendronate at the outer shell. The in vitro cytotoxic and antiangiogenic activity of the free drugs and micelles were the same. However, the micelles exhibited improved efficacy and safety profiles suggesting their potential use as bone-targeted anticancer and anti-angiogenic therapy for breast cancer bone metastases [199].

## 9. Problems Associated with Delivery Systems Used to Deliver Bisphosphonates 

Although the aforementioned delivery systems have been reported to be useful for the delivery of bisphosphonates and other bioactive agents, these systems also suffer from some limitations. Nitrogen-containing bisphosphonates such as alendronate contain primary amine groups. The conjugation of the amines to other drugs to form hybrid compounds compromises the therapeutic efficacy of the bisphosphonates. In some reports, conjugation of chemotherapeutic agents with bisphosphonates for enhanced selectivity towards bone metastases tumors resulted in compounds that did not exhibit antitumor effects [200,201]. In the incorporation of bioactive agents onto polymers to form polymer-drug conjugates, there are limitations such as slow release of the drug from the conjugates and low drug loading that can reduce the therapeutic efficacy of the incorporated drugs and inability to control the polymer synthesis resulting in low yield [202,203]. To overcome these limitations, the carriers are modified with functionalities so as to increase drug loading ability. Carbon nanotubes exhibit some shortcomings that limit their application in drug delivery such as their biosafety, which has been controversial, and there is a pressing need to investigate their safety in long term application. The application of liposomes for the encapsulation of bioactive agents is usually associated with low degrees of drug encapsulation and uncontrolled rates of drug release. To overcome these limitations, the method of drug attachment is usually modified followed by modulating lipid compositions, charges of the liposomes, particle sizes and addition of artificial polymers [1]. Ferreira reported long circulating liposomes which were achieved by adjusting the aforementioned factors [204]. In addition, the biodegradability and biocompatibility of bioactive ceramics, i.e., HAP, are often inadequate, thus limiting their potential clinical application [205]. These drawbacks can be overcome by blending with natural and synthetic polymers or by carefully selecting composite materials which enhance the scaffold properties, thus providing controlled degradation [206].

## 10. Conclusion

Bisphosphonates are employed for the therapy of diseases associated with the bone and also used in conjunction with anticancer drugs for selected types of cancer therapy. However, bisphosphonates exhibit some pharmacological drawbacks which include poor solubility, low oral bioavailability and toxicity. To overcome these limitations, several delivery systems for the drug have been employed for targeted and controlled delivery of bisphosphonates with enhanced therapeutic effects. These systems are reported to enhance the overall therapeutic effectiveness of bisphosphonates such as improved bioavailability, reduced toxicity and effective delivery of bisphosphonates to the specific target. However most of these systems have only been evaluated in vitro and in vivo and there is a pressing need for these systems to undergo clinical trials to ascertain its effectiveness for cancer therapeutic and other pathologies. Based on the research trends thus far, there is no doubt that some of these systems containing bisphosphonates will in the future reach clinical trials.

## Figures and Tables

**Table 1:** Polymer delivery systems.

Drug/Formulation	Carrier	Administration	Intended Application	Status	References
Neridronic	Polyamidoamine	-	Cancer	-	[120]
Bisphosphonates + curcumin	Polyamidoamine	-	Cancer	-	[120]
Bisphosphonates	Poly-hydroxy-aspartamide	-	Bone diseases	in vivo	[121]
Alendronate	poly(d,l-lactide-*co*-glycolide) (PLGA)	-	Bone diseases	in vitro	[122]
Alendronate	*N*-(2-hydroxypropyl) methacrylamide copolymer	Intravenous	Bone diseases	in vivo	[123]
Bisphosphonate	polyethylenglycol (PEG)	Intravenous	Bone diseases	in vivo	[124]
Bisphosphonate	polyglutamic acid (PGA)	Intravenous	Bone diseases	in vivo	[124]
Bisphosphonate	polylactic acid (PLA)	Intravenous	Bone diseases	in vivo	[124]
Bisphosphonate	polylactic-*co*-glycolic (PLGA)	Intravenous	Bone diseases	in vivo	[124]
Bisphosphonate	poly(lactide-*co*-glycolide)	Intravenous	Bone diseases	in vivo	[124]
Bisphosphonate	poly(d,l-lactide-*co*-glycolide) (PLA/PLGA)	Intravenous	Bone diseases	in vivo	[124]
Bisphosphonate	poly(hydroxyalkylmethaacrylamide)	Intravenous	Bone diseases	in vivo	[124]
Bisphosphonate	polyglycerol, a polyamidoamine (PAMAM)	Intravenous	Bone diseases	in vivo	[124]
Bisphosphonate	polyethylenimine (PEI)	Intravenous	Bone diseases	in vivo	[124]
Alendronate	poly[*N*-(2-hydroxypropyl) methacrylamide]	-	Bone diseases	in vitro	[125]

**Table 2:** Hydrogel delivery systems.

Drug/Formulation	Carrier	Administration	Intended Application	Status	References
Bisphosphonates	Acrylamide + gum acacia	-	Bone Treatment	-	[133]
Bisphosphonates	Hyaluronic acid hydrogel	-	Bone regeneration	in vitro	[134]
Risedronate sodium	Sodium alginate	-	Bone Treatment	-	[135]

**Table 3:** Bioceramic delivery systems.

Drug/Formulation	Bioceramic	Administration	Intended Application	Status	References
Alendronate	Mesoporous silica-based materials	-	Bone Treatment	-	[153]
Bisphosphonates	Hydroxyapatite (HA)	-	Bone graft substitute	in vitro	[154]
Zoledronic acid	HA	-	Bone graft substitute	in vitro	[156]
Zoledronic acid	Calcium phosphate (80% tricalcium phosphate, 20% HA)	-	Bone graft substitute	in vitro	[156]

**Table 4:** Hybrid Compounds.

Drug/Formulation	Administration	Intended Application	Status	References
LLP2A-Ale	Intravenous	Bone diseases	in vivo	[158]
Bisphosphonates + folic acid	-	Bone regeneration	in vitro	[159]
Bisphosphonate + Methotrexate	-	Osteosarcoma	in vitro	[160]
Bisphosphonate + gemcitabine	Intravenous	Bone metatases	in vivo	[161]
Bisphosphonate + platinum complexes	-	Bone Treatment	in vitro	[162]

**Table 5:** Carbon-based material delivery systems.

Drug/Formulation	Delivery System	Administration	Intended Application	Status	References
Bisphosphonates	Carbon nanotubes	-	Osteosarcoma	-	[171]
Bisphosphonate-fullerenes C_60_(OH)_16_AMBP	Fullerene	-	Bone mineralization	in vitro	[172]

**Table 6:** Liposome delivery systems.

Drug/Formulation	Delivery System	Administration	Intended Application	Status	References
Bisphosphonates	Liposome	-	Anticancer	in vitro	[185]
Bisphosphonates	Liposome	Intravenous	Treatment of stenotic coronary disease	in vivo	[187]
Clodronate	Liposome	Intravenous	Treatment of the spleen	in vivo	[188]
Bisphosphonate + PLAD	Liposome	-	Anticancer	in vivo	[189]

**Table 7:** Micelle delivery systems.

Drug/Formulation	Delivery System	Administration	Intended Application	Status	References
Bisphosphonate (thiolBP) + distearoylphospho-ethanolamine-polyethylene glycol	Micelle	-	Bone tissue engineering	in vitro	[197]
Doxorubicin-poly (ethylene glycol)-alendronate	Micelle	-	Bone cancer	in vitro	[198]
